# Associations among stressors, perceived stress, and psychological distress in nursing students: a mixed methods longitudinal study of a Hong Kong sample

**DOI:** 10.3389/fpsyg.2023.1234354

**Published:** 2023-08-17

**Authors:** Winnie Lai Sheung Cheng, Peggy Pik Kei Chow, Florence Mei Fung Wong, Mandy Man Ho

**Affiliations:** ^1^School of Health Sciences, Caritas Institute of Higher Education, Tseung Kwan O New Town, Hong Kong SAR, China; ^2^School of Nursing, Tung Wah College, Kowloon, Hong Kong SAR, China; ^3^School of Nursing, The University of Hong Kong, Pokfulam, Hong Kong SAR, China

**Keywords:** nursing students, stress, psychological distress, stressors, coping, mixed methods, longitudinal study

## Abstract

**Background:**

Nursing students are at risk for high-stress levels and psychological distress. Limited longitudinal studies have been conducted examining factors associated with stress levels and psychological distress of nursing students in their course of study.

**Purpose:**

The purpose of this study was to examine the levels of stress and corresponding stressors, particularly those predicting psychological distress, among nursing students over their 5 years of study.

**Methods:**

A longitudinal design, using questionnaires and focus group interviews of a single cohort of nursing students in Hong Kong and following them over their 5 years of training. The Stressors in Nursing Students Scale-Chinese version and the Chinese version of General Health Questionnaire-12 were used to assess stress levels and psychological distress, respectively.

**Results:**

Ninety-seven participants completed the questionnaires 5 times. Quantitative findings revealed that the overall stress levels of the nursing students increased over 5 years (from mean = 3.08 to 3.33), with the highest levels in the second wave (mean = 3.33). Nursing students experienced higher stress during years 2 (*p* = 0.006) and 4 (*p* = 0.037). Psychological distress was the highest in year 3 (sum score = 18.47) (*p* = 0.002) but declined from year 4 (*p* < 0.001). Thematic analysis revealed that academic performance issues, coping challenges, unfavorable learning environments, relationships were identified as the stressors. However, nursing students also used positive coping strategies to pursue success and seek support.

**Conclusion:**

This study suggests that the year of study is a significant predictor of stress levels among nursing students, especially during the first and senior years due to heavy academic workload. Psychological distress was observed among nursing students, and those who worked more part-time jobs tended to report higher levels of distress. The junior year was associated with higher levels of distress related to financial and time-related stress, while academic and personal problems were more prevalent during the senior year.

## 1. Introduction

Nursing is a stressful occupation (Woo et al., 2020; Babapour et al., 2022). Nursing students, who perform the same tasks as qualified nurses, shared the problem of stress during nurse training (Li and Hasson, 2020). The stress in nursing students may affect the relationship with the patients in the clinical practice. Experiencing high levels of stress and using ineffective coping strategies could compromise academic achievement, potentially leading to attrition from nursing study programs. This is particularly concerning given the already high attrition rates among nursing students globally (25% in Britain, 24.5% in Australia, 50% in the US, and 15–20% in Hong Kong, according to various studies) (Chan et al., 2019). In addition, chronic stress may lead to psychological and physiological ill-health (Rohleder, 2019; Knowles et al., 2020; Matud et al., 2020). Ineffective coping with stress may lead to psychological distress (Li et al., 2009; Collin et al., 2020), which is commonly associated with depression, anxiety, and social dysfunction. In a cross-sectional study, Cheng et al. (2022) reported that seeking support, problem-solving, and venting were the most common coping types used by the final year nursing students in Hong Kong; those nursing students who tended to use such coping strategies reported less psychological distress. Hence, assessment of various determinants of stress and distress is essential to suggest suitable remedial measures for stress mitigation.

Several researchers have identified the common stressors in nursing students as arising from clinical learning, academic study, social pressures and personal life (Ahmed and Mohammed, 2019; Aloufi et al., 2021; Chaabane et al., 2021). Most of these researchers used a cross-sectional design in looking at stress levels of nursing students in different years. These existing cross-sectional studies can, indeed, tell us about the association between variables; however, they do not tell us about time-varying factors. Both information about the temporal order of events causing these associations and inference of causality is limited, and it has been impossible to examine how stressors prospectively associate with the development of stress and psychological distress in nursing students.

Only a few studies have investigated the sources of stress throughout an entire nursing programme of study using longitudinal designs (Burnard et al., 2008; Mazalová et al., 2022). Importantly, these studies did not report which variables could be used to predict levels of stress and psychological distress. A longitudinal study conducted in five countries revealed that nursing students worldwide do share common stressors, such as academic and clinical elements, but individual cultural features relating to stress throughout their course of study also exist (Burnard et al., 2008). As in other countries, in Hong Kong, moderate to high stress levels among nursing students have been reported in several cross-sectional studies (Ching et al., 2020; Xie et al., 2022). This paper examines both stress levels and psychological well-being in nursing students over the course of their training using a longitudinal study design. Longitudinal study design allows us to assess stress-related factors across the course of study. Moreover, this design allows us to use time as a variable; it provides temporal changes of variables in individuals, and it yields order of changes which can help provide a stronger inference of causality (Avey et al., 2008). In addition, longitudinal studies provide data that can be analyzed using repeated measures; thus, statistical inference can be made with fewer subjects compared with cross-sectional designs.

### 1.1. Theoretical framework

Stress is a state of psychological arousal that occurs when external demands exceed a person’s ability to cope (Lazarus and Folkman, 1984). It can have either positive or negative effects, depending on different situation and individual’s response (Laal and Aliramaie, 2010). High level of stress and ineffective coping skills could be a barrier to achievement (Singh et al., 2011). The transactional model of Lazarus and Folkman (1984) proposes that stress is a result of the interaction between the individual and the environment. This model has guided numerous studies exploring the interplay between coping strategies and stress (LeSergent and Haney, 2005; Watson et al., 2008; Lin et al., 2010). Coping strategies are often utilized after individuals have appraised a situation as threatening to their well-being (Lazarus and Folkman, 1984), and the choice of coping strategies depends on how individuals perceive a situation (Bhurtun et al., 2019; Onieva-Zafra et al., 2020). Nursing students who experience high levels of stress during their course of study and employ ineffective coping strategies are at a higher risk of developing psychological distress (Li et al., 2009; Salvarani et al., 2020). Coping strategies can potentially moderate the impact of stress on psychological distress. As nursing students are likely to encounter personal, academic, and work-related stressors during their course of study, it is critical to investigate the associations among these factors for developing effective interventions to reduce psychological distress. Guided by the transactional model, the specific aims of this study are to investigate the impact of stressors, stress, and coping strategies on psychological distress.

The specific objectives of the present study were therefore: (1) to examine the sources of stress and change in stress levels experienced by nursing students during their five-year academic study; (2) to ascertain the relationships between stressors, coping strategies, stress and psychological distress; and (3) to identify the stressful experience(s) during their nursing programme.

## 2. Methods

### 2.1. Design

This study used a prospective, longitudinal design; it included both a quantitative survey and qualitative focus group interviews. The manuscript was written according to the STROBE guideline for cohort studies (Tong et al., 2007) and COREQ for qualitative studies (Von Elm et al., 2007).

### 2.2. Participants

The study was conducted in a private higher educational institution offering baccalaureate nursing programme in Hong Kong. The programme is provided over 5 years; nursing students were required to meet the theoretical (at least 1,250 theoretical hours) and clinical practice (at least 1,400 h) requirements at specific standards throughout the study [The Nursing Council of Hong Kong (NCHK), 2023]. All 242 first-year full-time nursing students enrolled during the fall of 2014 in a five-year baccalaureate nursing programme were recruited to minimize sample error. For regression analysis within-group, a sample size of 48 participants was adequate to yield a statistical power of 0.8 with an effect size of 0.25 and with an α value of 0.05 (bidirectional hypothesis) measuring four times (Faul et al., 2007).

### 2.3. Procedure

The longitudinal survey began in 2014; questionnaires were administered once per year through 2018 (5 years, total). Questionnaires were administered during a lecture class which was offered to the same cohort, each year. In the first year, a total of 242 questionnaires were administered; 164 were returned in the first wave (response rate 68%), 108 in the second wave (response rate 45%), 89 in the third wave (response rate 37%), 134 in the fourth wave (response rate 55%), and 109 in the fifth wave (response rate 45%). The participants were lost to follow-up in each stage due to absence and course drop-out. Ultimately, 97 questionnaires were analyzed from the participants who consistently responded throughout all 5 years of study. Focus group interviews were conducted in the fifth year to explore the cumulative lived experience of stress. The ethical committee of the college where the study was conducted approved the study. Informed consent was obtained from participants prior to data collection. The participants were informed about the purpose of the study and their rights before beginning the anonymous survey through an information sheet.

### 2.4. Measurements

We used the Stressors in Nursing Students Scale-Chinese version (SINS-CN) (Wong, 2014) to assess the five dimensions of stress with 43 items: clinical learning (17 items), an example of which includes “patients’ attitude towards me”; academic study (4 items), an example of which includes “having too much to learn”; finance and time (7 items), an example of which includes “having no time for entertainment”; confidence (11 items), an example of which includes “not being sure what is expected from placement”; and personal problems (4 items), an example of which includes “personal health problem.” The scale was scored using a five-point Likert-type scale (1 = “not at all stressful” to 5 = “extremely stressful”). It is a 43-item scale originally developed by Deary et al. (2003). The SINS-CN was cross-culturally validated with nursing students in Macao (SAR of China), where the cultural differences between Macao and Hong Kong are relatively minor. The content validity index (CVI) was 0.89; principal component analysis yielded a 5-component structure with 43 items and accounted for 60.28% of the variance; factor loadings of the five dimensions ranged from 0.42 to 0.79; Cronbach’s alpha was 0.96 for the overall scale (Wong, 2014). Cronbach alpha ranged from 0.864 to 0.946 in this study.

We included the Chinese version of the General Health Questionnaire-12 (C-GHQ-12) after we had obtained funding in 2015 to extend the original cross-sectional study to a longitudinal study. The GHQ-12 is a widely used screening tool with 83.4% sensitivity and 76.3% specificity (Goldberg et al., 1997). This 12-item scale uses four-point Likert scoring from 0 to 3 (0 = Not at all, 1 = occasionally, 2 = sometimes, or 3 = always). An example item is “Have you recently been able to concentrate on what you are doing?.” Total scores range from 0 to 36, with scores greater than 15 indicating psychological distress. Cronbach alpha ranged from 0.698 to 0.895 in this study.

Demographic information including gender, age, educational attainment, marital status, religion, and coping behaviors was also collected. The Chinese version of Brief Cope Inventory (Brief COPE) was used to assess the participants’ coping behavior (Yuan et al., 2017). The Chinese version of Brief COPE has 14 subscales each of which consists of two items. The subscales are: self-distraction, active coping, denial, substance use, use of emotional support, use of instrumental support, behavioral disengagement, venting, positive reframing, planning, humor, acceptance, religion, and self-blame (Carver, 1997).

### 2.5. Focus group interview

We used focus group interviews to explore the feelings and personal experience of nursing students during their academic life. Purposive sampling was used to recruit the participants. In view of the sensitive and personal nature of the interviews, the participants we recruited all know each other so that they could relate to each other and feel comfortable in disclosing their feelings (Krueger and Casey, 2000). We initially planned to interview three focus groups until we had enough information or until the data was saturated theoretically (Krueger and Casey, 2000). Ultimately, two focus grouped were recruited; the first focus group had four students, while the other had five. The interviews were organized at the study institution at a time and date convenient for participants and researchers. Participants were over-recruited by 20% (i.e., eight students recruited for the six spots) in anticipation of the recruitment difficulty due to their engagement in clinical duty schedule. Interviews were moderated by the principal researcher (WL-SC) who is experienced in conducting research interviews. The moderator facilitated an environment in which the participants felt relaxed and connected. The interview questions included: (1) Was there anything stressful during your study? (2) What was that experience? (3) How did that stressful experience affect you? (4) How did you cope with that experience? These open-ended questions guided the discussion to focus on the topic. The moderator followed the question guide and also asked questions to obtain more in-depth information, including actions taken to mitigate stress and what the school had done to reduce stress. Participants were encouraged to speak freely and express their true feelings. During the interviews, a research assistant took notes, recording any special expressions and non-verbal interactions and recording which statements were made by which participant. The interviews were audio-recorded with consent. Each interview lasted approximately 1.5 h. The data were saturated at the second focus group interview.

## 3. Data analysis

### 3.1. Quantitative analysis

Data were managed and analyzed by SPSS 23 for Windows. Descriptive statistics were used to summarize the characteristics of the samples and stress and psychological distress outcomes. Normality of data was assessed using the Shapiro–Wilk test and measures of skewness. Pearson’s correlation and Chi-square tests were used to test the relationships between variables. The data set was longitudinal observation with repeated measures. Missing data were completed with series mean. As longitudinal data, the outcome variables involved statistical concerns with regard to heteroscedasticity and heterogeneity. To address the above challenges, we used multilevel mixed effects linear regression models (Generalized linear mixed models) (GLMM; SPSS Version 22) to explore separately the relationships between stress or psychological distress and stressors, coping strategies, and demographic characteristics. GLMMs allow response variables to vary for different distributions (Bolker et al., 2009). Thus, it can identify temporal autocorrelations and unobserved heterogeneity through modeling group-specific variability in trajectories over time and subject-specific differences (Qureshi and Fang, 2010). Using GLMMs has advantages with longitudinal data in which repeated measures are nested within participants because GLMMs can account for dependence of residuals due to covariance between the levels in the repeated measurements (McCulloch and Searle, 2001). Also, GLMMs can model both fixed and random effects; thus, it can model change/growth in individuals over time (McCulloch and Searle, 2001). Fixed effects refer to estimates where only one intercept and one slope are fitted to the data. In this way, both the initial level of stress and psychological distress and the rate of change over time are modelled to be equal for all participants. In addition, GLMMs are robust to loss to follow-up under the missing at random assumption, since some participants were lost to follow up at the end of the study and the data were unbalanced.

Random effects analyses were performed to examine the within-individual error covariance. This method improves model precision and statistical inferences. For our data, there are five possible random effects: random intercept, random coefficients of demographic characteristics, coping behaviours, stressors, and study years. We ran five random effect models to fit the data of the stress level and psychological distress separately. We chose the most appropriate model among the five random effect models of each target using model fitting criteria (AIC, Akaike Information Criterion and BIC, Bayesian Information Criterion) (Akaike, 1980). Models with the smallest AIC and BIC values were chosen. In the GLMM analysis for stress levels as target, we included age as random effect, while in the GLMM analysis for psychological distress, we included study year as random effect. The fixed effects were demographic characteristics (significant variables were included for analysis: age for the regression analysis on stress levels; part-time job for the regression analysis on psychological distress levels), the study years, stressors, coping behaviors, and the two-way interactions of stressors with study year. All statistical tests were two-tailed, and variables were considered significant at *p* = 0.05.

### 3.2. Qualitative analysis

Each focus group was audio-recorded. Narrative data were transcribed verbatim by two trained research assistants independently, and further reviewed by a third research assistant to ensure accuracy. Thematic analysis was used to analyze the qualitative data (Braun and Clarke, 2014). The six steps of thematic analysis are: familiarization with the data; generating codes; searching for themes; reviewing the themes; defining and naming the themes; producing the report (Kiger and Varpio, 2020). Independent thematic analysis was performed by two authors (FW and PC) to ensure reliability of the data interpretations. Discrepancies and inconsistencies were checked by the third author (WL-SC). We examined all quotes for recurrent terms, which were then systematically identified across the data set and coded and grouped into sub-themes and further categorized into main themes (Krueger and Casey, 2000). Each quotation was coded with a letter and a number. For example, A1 refers to participant A of the first focus group. B2 refers to the participant B of the second focus group. Qualitative rigor was maintained through checking, transcript confirmation and inter-rater analysis. Interrater agreement was 0.80 and 0.90 for themes and sub-themes, respectively. We discussed inconsistencies until agreement on the overall themes and sub-themes was reached.

## 4. Results

### 4.1. Quantitative results

In this study, the average age of the nursing students was 18.82 years (SD = 1.275), with 75.3% (*N* = 73) being female. All were single. Approximately 77% (*N* = 75) had part-time jobs. Table 1 presents the mean scores and the standard deviation for the stress levels, sources of stress, and psychological distress over the years. Overall stress levels ranged from mean = 3.08 to 3.33. Stress arising from academic study scored the highest (mean = 3.84). Regarding the C-GHQ-12, overall scores declined from sum = 18.15 in year 2 to 15.33 in year 5; i.e., psychological distress tended to decline over the years. The coping behaviors used were mostly positive: use of information (mean = 3.1, SD = 0.45), emotional support (mean = 3.0, SD = 0.55), venting (mean = 3.0, SD = 0.50), and planning (mean = 2.9, SD = 0.43). However, the use of negative coping strategies significantly correlated with the increase of stress levels. Stress level was positively, significantly correlated with denial, behavior disengagement, humor, and self-blame, whereas behavior disengagement, venting, and self-blame were negatively correlated with psychological distress. Table 2 shows the correlations between stress, psychological distress levels and coping behaviors over the years.

**Table 1:** Descriptive statistics for the stress levels, stressors and psychological distress over the years (*N* = 97).

	2014		2015		2016		2017		2018	
	Mean	SD	Mean	SD	Mean	SD	Mean	SD	Mean	SD
Overall stress levels	3.08	0.47	3.33	0.42	3.21	0.59	3.21	0.37	3.13	0.42
Stressors										
Clinical learning (1–5)	2.70	0.82	3.34	0.71	3.18	0.65	3.34	0.42	3.26	0.49
Confidence (1–5)	3.04	0.57	3.23	0.56	3.23	1.05	3.10	0.47	2.30	0.53
Finance and Time (1–5)	3.11	0.70	3.11	0.70	3.16	0.79	3.28	0.58	3.14	0.66
Academic study (1–5)	3.84	0.56	4.14	0.54	3.70	0.60	3.54	0.44	3.56	0.52
Personal problems (1–5)	2.71	0.66	2.80	0.63	2.79	0.68	2.78	0.53	2.69	0.59
Psychological distress (0–36)	–	–	18.15	2.35	18.47	3.83	17.65	2.72	15.33	2.00

**Table 2:** Correlation between coping strategies and stress levels and psychological distress by years.

	Brief COPE-C (subscales)													
	1	2	3	4	5	6	7	8	9	10	11	12	13	14
Stress levels														
2014 (T1)	0.010	0.117	0.263^**^	0.143	0.076	0.098	0.060	0.033	0.082	−0.069	0.211^*^	−0.009	−0.068	0.137
2015 (T2)	−0.087	−0.019	0.234^*^	0.119	−0.071	−0.001	0.054	−0.107	−0.044	−0.111	0.125	−0.084	−0.075	0.325^**^
2016 (T3)	0.008	−0.037	0.220^*^	0.054	0.030	0.031	0.171	−0.078	0.008	−0.095	0.010	−0.069	0.036	0.201^*^
2017 (T4)	0.008	−0.018	0.111	−0.025	0.053	0.081	0.197	0.047	−0.103	−0.090	0.072	−0.207^*^	0.012	0.298^**^
2018 (T5)	0.109	0.100	0.425^***^	0.096	−0.098	−0.090	0.426^***^	−0.054	−0.124	−0.114	0.310^**^	−0.176	0.007	0.565^***^
Psychological distress														
2015 (T2)	0.066	−0.086	0.113	0.080	−0.049	−0.025	0.101	−0.034	−0.079	−0.176	−0.046	−0.163	−0.017	0.105
2016 (T3)	−0.047	0.026	−0.139	−0.182	−0.022	0.015	−0.224^*^	−0.041	0.156	0.076	−0.092	0.086	−0.033	−0.332^***^
2017 (T4)	0.066	−0.076	0.173	0.183	−0.006	0.066	0.142	0.099	−0.179	−0.129	0.116	−0.187	−0.084	0.243^**^
2018 (T5)	−0.196	−0.067	0.032	−0.177	−0.011	−0.085	−0.004	−0.245^*^	0.144	−0.102	−0.024	−0.024	−0.120	−0.165

*p ≤ 0.05; **p ≤ 0.01; ***p ≤ 0.001. Numbers 1–14 refer to the subscales of the Brief Cope Inventory-Chinese version (Brief COPE-C), Where, 1 = self-distraction; 2 = active coping; 3 = denial; 4 = substance abuse; 5 = emotional support; 6 = use of information; 7 = behaviour disengagement; 8 = venting; 9 = positive reframing; 10 = planning; 11 = humour; 12 = acceptance; 13 = religion; 14 = self-blame.

For the associations between stressors, stress and psychological distress, all stressors were positively related to stress levels in years 1–5. For psychological distress, the stressors related to confidence, finance and time, academic study, and personal problems were positively correlated with psychological distress in year 2. In year 3, all the stressors were negatively correlated with psychological distress, whereas in year 4, all stressors were positively correlated with psychological distress (Table 3).

**Table 3:** Correlation between stressors and stress levels and psychological distress by years.

	Stressors				
	Clinical	Confidence	Finance and time	Academic study	Personal problem
Stress levels					
2014 (T1)	0.596^***^	0.869^***^	0.770^***^	0.599^***^	0.726^***^
2015 (T2)	0.230^*^	0.564^***^	0.606^***^	0.357^***^	0.483^***^
2016 (T3)	0.761^***^	0.814^***^	0.815^***^	0.754^***^	0.770^**^
2017 (T4)	0.774^***^	0.871^***^	0.675^***^	0.689^***^	0.785^***^
2018 (T5)	0.407^***^	0.327^**^	0.254^*^	0.248^*^	0.300^**^
Psychological distress					
2015 (T2)	0.121	0.440^***^	0.324^**^	0.321^**^	0.342^**^
2016 (T3)	−0.369^***^	−0.401^***^	−0.515^***^	−0.415^***^	−0.544^***^
2017 (T4)	0.448^**^	0.531^**^	0.365^**^	0.506^**^	0.543^**^
2018 (T5)	−0.077	−0.087	−0.208^*^	−0.070	−0.150

*p ≤ 0.05; **p ≤ 0.01; ***p ≤ 0.001.

As shown in Table 4, year of study (year 2 and 4), stressors related to clinical study, confidence, finance and time, academic study, and personal problems were significant positive predictors of stress. In year 1, the stressor related to personal problems was a positive predictor of stress, while in year 3, confidence was a negative predictor.

**Table 4:** Estimates of dependence of stress score and psychological distress score on demographic, year of study, stressors, coping strategies, and interaction terms between year of study and stressors from generalized linear mixed model.

	Stress						Psychological distress					
Effect	Estimate (β)	SE	*t* value	*p*-value	95% CI		Estimate (β)	SE	*t* value	*p*-value	95% CI	
					*UL*	*LL*						
Intercept	0.137	0.0215	6.348	0.000	0.094	0.179	3.178	0.1880	16.904	0.000	2.807	3.548
Demographic characteristics^a^												
Age	−0.001	0.0007	−1.125	0.261	−0.002	0.001	–	–	–	–	–	–
Part-time job: No	–	–	–	–	–	–	0.045	0.0308	1.466	0.144	−0.016	0.106
Less than 3 h/week	–	–	–	–	–	–	0.025	0.0320	0.781	0.435	−0.038	0.088
4–10 h/week	–	–	–	–	–	–	0.049	0.0266	1.846	0.066	−0.003	0.102
More than 10 h/week	–	–	–	–	–	–	0.057	0.0261	2.183	0.030	0.006	0.108
Irregular working hrs	–	–	–	–	–	–	0^b^	.	.	.	.	.
Year of study^a^												
Year 1	−0.014	0.0164	−0.870	0.385	−0.047	0.018	–	–	–	–	–	–
Year 2	0.047	0.0169	2.748	0.006	0.013	0.080	−0.673	0.2118	−3.178	0.002	−1.091	−0.256
Year 3	0.028	0.0176	1.563	0.119	−0.007	0.062	0.687	0.2153	3.194	0.002	0.263	1.112
Year 4	0.034	0.0160	2.095	0.037	0.002	0.065	−1.135	0.2352	−4.824	0.000	−1.598	−0.671
Year 5	0^b^	.	.	.	.	.	0^b^	.	.	.	.	.
Self-distraction	0.001	0.0022	0.551	0.582	0.001	0.0022	−0.008	0.0170	−0.478	0.633	−0.042	0.025
Active coping	0.000	0.0024	0.182	0.856	0.000	0.0024	0.000	0.0265	−0.014	0.989	−0.053	0.052
Denial	0.001	0.0024	0.516	0.606	0.001	0.0024	0.019	0.0277	0.697	0.487	−0.035	0.074
Substance-abuse	0.001	0.0019	0.499	0.618	0.001	0.0019	−0.016	0.0158	−1.022	0.308	−0.047	0.015
Emotional support	0.001	0.0025	0.201	0.840	0.001	0.0025	−0.014	0.0191	−0.715	0.475	−0.051	0.024
Use of information	−0.002	0.0028	−0.834	0.405	−0.002	0.0028	0.022	0.0202	1.080	0.281	−0.018	0.062
Behaviour disengagement	−0.002	0.0020	−1.133	0.258	−0.002	0.0020	0.002	0.0232	0.072	0.942	−0.044	0.047
Venting	−0.001	0.0019	−0.497	0.620	−0.001	0.0019	−0.012	0.0183	−0.667	0.506	−0.048	0.024
Positive framing	−0.001	0.0021	−0.567	0.571	−0.001	0.0021	0.023	0.0159	1.466	0.144	−0.008	0.055
Planning	0.001	0.0027	0.282	0.778	0.001	0.0027	−0.024	0.0233	−1.010	0.313	−0.070	0.022
Humour	0.000	0.0017	0.266	0.791	0.000	0.0017	0.024	0.0181	1.303	0.194	−0.012	0.059
Acceptance	−0.003	0.0025	−1.286	0.199	−0.003	0.0025	−0.029	0.0251	−1.136	0.257	−0.078	0.021
Religion	0.000	0.0012	−0.240	0.810	0.000	0.0012	0.004	0.0128	0.330	0.742	−0.021	0.029
Self-blame	0.002	0.0018	1.088	0.277	0.002	0.0018	0.003	0.0168	0.173	0.863	−0.030	0.036
Clinical	0.070	0.0056	12.535	0.000	0.059	0.081	−0.030	0.0876	−0.344	0.731	−0.203	0.142
Confidence	0.065	0.0058	11.229	0.000	0.054	0.077	0.043	0.0852	0.510	0.611	−0.125	0.211
Finance and time	0.067	0.0030	22.536	0.000	0.061	0.073	−0.083	0.0410	−2.037	0.043	−0.164	−0.003
Academic study	0.063	0.0035	17.986	0.000	0.056	0.070	−0.033	0.0419	−0.780	0.436	−0.115	0.050
Personal problem	0.059	0.0033	17.763	0.000	0.053	0.066	−0.038	0.0442	−0.854	0.394	−0.125	0.049
Stressors and years of study interaction^a^												
Clinical *[year 1]	−0.003	0.0059	−0.540	0.590	−0.015	0.008	–	–	–	–	–	–
Clinical *[year 2]	−0.006	0.0059	−0.950	0.343	−0.017	0.006	0.028	0.0929	0.305	0.761	−0.155	0.211
Clinical *[year 3]	−0.005	0.0071	−0.667	0.505	−0.019	0.009	0.029	0.0961	0.300	0.764	−0.161	0.218
Clinical *[year 4]	−0.010	0.0071	−1.362	0.174	−0.024	0.004	−0.016	0.1170	−0.135	0.893	−0.246	0.215
Clinical *[year 5]	0^b^	.	.	.	.	.	0^b^	.	.	.	.	.
Confidence *[year 1]	0.004	0.0071	0.564	0.573	−0.010	0.018	–	–	–	–	–	–
Confidence *[year 2]	−0.008	0.0067	−1.150	0.251	−0.021	0.005	0.034	0.0951	0.354	0.724	−0.154	0.221
Confidence *[year 3]	−0.017	0.0063	−2.762	0.006	−0.030	−0.005	−0.030	0.0898	−0.332	0.740	−0.207	0.147
Confidence *[year 4]	−0.002	0.0070	−0.257	0.798	−0.016	0.012	0.001	0.0986	0.007	0.995	−0.194	0.195
Confidence *[year 5]	0^b^	.	.	.	.	.	0^b^	.	.	.	.	.
Finance and time*[year 1]	−0.003	0.0041	−0.678	0.498	−0.011	0.005	–	–	–	–	–	–
Finance and time*[year 2]	−0.005	0.0036	−1.468	0.143	−0.012	0.002	0.116	0.0475	2.450	0.015	0.023	0.210
Finance and time*[year 3]	−0.002	0.0046	−0.466	0.642	−0.011	0.007	−0.029	0.0503	−0.577	0.565	−0.128	0.070
Finance and time*[year 4]	−0.004	0.0037	−1.153	0.250	−0.011	0.003	0.089	0.0509	1.754	0.081	−0.011	0.189
Finance and time*[year 5]	0^b^	.	.	.	.	.	0^b^	.	.	.	.	.
Academic study *[year 1]	−0.001	0.0046	−0.231	0.817	−0.010	0.008	–	–	–	–	–	–
Academic study *[year 2]	0.001	0.0046	0.282	0.778	−0.008	0.010	0.044	0.0494	0.889	0.375	−0.053	0.141
Academic study *[year 3]	0.006	0.0059	1.096	0.274	−0.005	0.018	−0.082	0.0617	−1.336	0.183	−0.204	0.039
Academic study *[year 4]	0.005	0.0049	1.002	0.317	−0.005	0.015	0.174	0.0721	2.415	0.017	0.032	0.316
Academic study *[year 5]	0^b^	.	.	.	.	.	0^b^	.	.	.	.	.
Personal problem*[year 1]	0.009	0.0045	2.054	0.041	0.000	0.018	–	–	–	–	–	–
Personal problem*[year 2]	0.003	0.0040	0.707	0.480	−0.005	0.011	0.039	0.0529	0.745	0.457	−0.065	0.144
Personal problem*[year 3]	0.007	0.0051	1.406	0.160	−0.003	0.017	−0.032	0.0639	−0.505	0.614	−0.158	0.094
Personal problem*[year 4]	0.001	0.0043	0.202	0.840	−0.008	0.009	0.151	0.0626	2.420	0.016	0.028	0.275
Personal problem*[year 5]	0^b^	.	.	.	.	.	0^b^	.	.	.	.	.
Fit statistics												
-2LogL	−2339.736						−101.337					
AIC	−2325.477						−88.953					
BIC	−2297.129						−68.814					

AIC, Akaike Information Criterion; BIC, Bayesian Information criterion; CI, confidence interval; LL, lower limit; UL, upper limit.

a Main effect for stress score = age, year of study (1–5), stressors and year of study interaction (1–5); Main effect for psychological distress score = part-time job, year of study (2–5), stressors and year of study interaction (2–5).

b This coefficient is set to zero because it is redundant.

For psychological distress, part-time job employment with more than 10 h per week, year of study, and the stressors related to finance and time were the significant positive predictors (Table 4). In year 2, when the stressors interacted with year of study, finance and time positively predicted psychological distress. In year 4, academic study and personal problems positively predicted psychological distress.

### 4.2. Qualitative results

Nine participants (*N* = 9, 4 male, 5 female; mean age, 18.7 years, SD = 0.89) took part in the focus group interviews in the fifth year (Table 5). Thematic analyses of the focus group transcripts identified five themes: academic performance issues, unfavorable environment, coping challenges, relationships, and pursuit for success and support, with subthemes under each theme. The subthemes were assigned with numerical codes, and the corresponding frequencies were calculated.

**Table 5:** Characteristics of focus group participants.

Gender	Female	5
	Male	4
Year of study		5
Age	Mean	18.7
	SD	0.89

Under academic performance issues, the subthemes were ambiguous course information, fear of failing, heavy workload, lack of knowledge and confidence. The highest-ranked subthemes were fear of failing, heavy workload, and lack of knowledge and confidence, each with a frequency of 6.

Under unfavorable learning environment, the subthemes were lack of support and resources, and adaptation demand. The highest-ranked subtheme was lack of support and resources, with a frequency of 10.

Under coping challenges, the subthemes were financial burden, uncertainty about the future, lack of work-life balance, and health problems. The highest-ranked subtheme was health problems, with a frequency of 6.

Under relationships, the subthemes were desire for respect from faculty and clinical ward staff, and fear of disappointing parents. Both subthemes were ranked the same with a frequency of 3.

Under pursuit of success and support, the subthemes were help-seeking and self-reliance. The highest-ranked subtheme was help-seeking, with a frequency of 7. Table 6 presents the numerical codes and frequencies of the subthemes.

**Table 6:** The list of themes and sub-themes, the numerical code of each sub-theme, and their frequencies.

Themes	Sub-themes	Numerical code	Frequency
Academic performance issues	Ambiguous course information	1	3
	Fear of failing	2	6
	Heavy workload	3	6
	Lack of knowledge and confidence	4	6
Unfavourable learning environment	Lack of support and resources	5	10
	Adaptation demand	6	5
Coping challenges	Financial burden	7	4
	Uncertainty about future	8	2
	Lack of work-life balance	9	3
	Health problems	10	6
Relationships	Desire for respect from faculty and clinical ward staff.	11	3
	Fear of disappointing parents	12	3
Pursuit of success and support	Help-seeking	13	7
	Self-reliance	14	5

The following paragraphs present the themes and sub-themes. The representative quotations are highlighted in italics.

#### 4.2.1. Theme 1: academic performance issues

##### 4.2.1.1. Ambiguous course information

The categories under this sub-theme are the inconsistency of marking criteria and teaching approach among educators. The nursing students attributed their failure to these factors.

*I found it confusing, especially when it came to writing a care plan. There was no standard format; we were given Format A during lectures, Format B in tutorials, and then Format C in consultation sessions.* (D2)

*We had to follow different assessment requirements from different teachers. This made it difficult to keep track of what was expected of us … the marking guidelines were unclear, and I ended up failing the course. The grading scheme from Teacher A was different from Teacher B, which was inconsistent.* (C1)

##### 4.2.1.2. Fear of failure

Risk of dismissal from the nursing programme due to failure of exams was described as a major source of stress. The stress in achieving a pass grade for Non-Communicable Disease (NCD) courses and for skill tests was highlighted by students.

*I failed my clinical practicum, and I couldn’t stop thinking about the possibility of being deferred if I failed again. I was afraid that my GPA would fall below 2 in two semesters, which could result in dismissal.* (B2)

*During my clinical placement, I was extremely stressed while preparing for the skill test on administering medication. On the day of the exam, the patients that I had prepared for were unexpectedly discharged, and I had to find new cases to test on. This experience was so overwhelming that I burst into tears whenever I recalled it.* (B1)

##### 4.2.1.3. Heavy workload

Many nursing students described the number and nature of examinations, coursework, and clinical learning as a significant source of stress throughout their study. The demands of academic and clinical workload adversely affected their social, physical, and psychological well-being.

*… The clinical practicum schedule was very demanding, and I had to balance that with completing essays and preparing for exams at the same time …* (E1)

*I felt extremely stressed while working in the surgical ward. It was so busy that I didn’t have enough time to prepare for my clinical assessment or for a job interview with the hospital …* (B2)

*It was difficult to manage so many tasks at the same time. We needed to complete assignments, prepare for exams, and also work shifts, which left us with very little time … we had to balance our family and social lives. I also had trouble with sleep. Although I fell asleep easily, I woke up frequently and had trouble falling back asleep. It felt like I never really slept, and I felt tired every day. Every time I thought about the ward, I would think of the tasks waiting for me to complete. Even when I got back home, I would worry about unfinished tasks and fear that something might have been left incomplete.* (D2)

##### 4.2.1.4. Lack of knowledge and confidence

Lack of knowledge and confidence among nursing students can lead to negative emotions such as frustration, anxiety, and self-doubt. The nursing students felt overwhelmed by coursework and clinical placements, struggled to keep up with peers, and experienced shame or embarrassment about their perceived lack of skills. This can have personal and professional consequences for the nursing students and the healthcare system.

*I felt overwhelmed during my first clinical placement because I had no prior experience with the ward setting, and I was uncertain about the tasks that needed to be completed and how to perform them effectively. I continued to feel tense even after my clinical duty was over. When I returned home … and would worry about making mistakes.* (B1)

*The ward staff asked me why I was standing there doing nothing, and it was because I didn’t feel competent enough to provide patient care … after returning home, I constantly worried about what I might have missed during my shift and feared being blamed for any mistakes.* (D2)

*I understand that it’s my responsibility to learn, but I didn’t know how to approach it. I struggled with reading patient records due to the many new terms and concepts. It was overwhelming to encounter this new knowledge without a clear understanding of how to apply it to patient care …* (C2)

*I often felt pressured to keep up with the pacing of my fellow classmates, and I was afraid of falling behind …* (A2)

#### 4.2.2. Theme 2: unfavorable learning environment

##### 4.2.2.1. Lack of support and resources

This sub-theme pertains to the inadequacy of both human and material support, particularly in terms of guidance, assistance, and learning resources. Nursing students have highlighted that they lacked sufficient support from faculty and clinical mentors in terms of guidance and assistance. Additionally, they faced a shortage of learning resources and equipment, which impacted their learning experience.

*When I failed a course, I reached out to the school for advice. However, their response was too formal and lacked concrete advice. They simply told me to study harder, which was not particularly helpful. I had sought help because I was struggling and needed more guidance. Unfortunately, their response was vague and unhelpful, and I was unable to receive the assistance I needed …* (A1)

*… our lab sessions are only one hour long, and after the teacher’s demonstration, we are left with only 30 minutes to practice skills, which we have to share with 25 other students … there is often a shortage of materials, and we have to compete with other students for practice opportunities … booking time with the teacher for practice is also challenging, as it is often not available.* (C2)

*During my ward practice, I encountered staff who were not very enthusiastic about teaching or did not have enough time to spend with us due to the ward’s busyness.* (E1)

*During our skill test, the patient I had prepared for had already been discharged due to the high turnover of patients in the ward, leaving me feeling shocked and unprepared when I arrived for the test … the ward staff did not facilitate the process … … [additionally] I felt like I was being treated as a manpower, having to prepare for my test while also working as a helping hand in patient care.* (B1)

*It has been communicated to us that teachers are only available to answer our questions during office hours, from 9: 00 am to 5:00 pm, Monday to Friday. This limited availability can be challenging for students … we may have questions or concerns outside of these hours. We feel helpless in such situations and wonder if it would be possible for teachers to be more flexible with their availability.* (D1)

##### 4.2.2.2. Adaptation demand

The categories under this sub-theme pertain to the stress related to constantly adapting to new routine, cultures, and teaching styles during the clinical practice. The frequent change of clinical venues can contribute to psychological and physical ill health, as it can be demanding to adapt to new environments. Being in an unfamiliar environment in terms of the ward routine and culture during clinical learning is stressful for most nursing students.

*The change of resource person happened twice during my clinical practice. The sudden departure of the resource person made it difficult for me to adapt, and not knowing the teacher’s standards added an extra layer of challenge.* (A1)

*The clinical practice was stressful because we only stayed in each ward for two weeks. This meant that we had to constantly adapt to new routines, cultures, and skills. By the time we had finally gotten the hang of things, we would be moved to a new ward.* (C2)

*I was apprehensive about the new ward, unsure of how the staff would treat the students.* (D1)

#### 4.2.3. Theme 3: coping challenges

##### 4.2.3.1. Financial burden

The categories under this sub-theme focus on the worry of nursing students who are financially burdened by the high cost of tuition and the uncertainty of finding a job after graduation.

*I had already spent HK$45,000 on tuition fees for the program, and I knew it would be difficult to pay back the loan if I couldn’t find a job.* (C1)

*I was worried about how I would repay the student loan. It would be a huge financial burden on my parents, and I needed to support the family as well.* (B2)

##### 4.2.3.2. Uncertainty about future

Uncertainty about the future can be a major source of stress and anxiety for nursing students. They worry about not being able to find a job after graduation, and about not being able to pay back their student loans.

*The thought of job interviews was frightening because they were demanding … [additionally] the thought of not finding a nursing job after graduation was devastating. …* (A1)

*I was so lost and uncertain about my future. I didn’t know if I would be able to find a job …* (B2)

##### 4.2.3.3. Lack of work-life balance

Heavy workloads can contribute to a lack of work-life balance, which can have a negative impact on the health of nursing students. This can lead to physical and psychological ill health, as well as difficulty maintaining relationships with family and friends.

*We had to work from Monday to Saturday, and if we had a Saturday afternoon shift, we had very little time to rest. I was assigned to have Saturday afternoon shifts for six consecutive weeks, which left me too tired to do anything for leisure for nearly six months. Spending time with family and friends became a luxury.* (E1)

*I failed a course, so I had to put in extra effort and time to study. I had less time to sleep and no time for entertainment.* (C2)

##### 4.2.3.4. Health problems

The nursing students experienced various health problems due to the stress associated with their studies. Trouble falling asleep, loss of appetite, and mental health issues such as depression, anxiety, and irritability are common health problems commonly experienced by nursing students due to the demands of their studies.

*Throughout the semester, I experienced poor sleep due to the fear of failing, irritability, weight loss due to a lack of appetite, and feelings of depression and worry.* (B2)

*After the skill test, my heart rate increased up to 160 for some days, and I couldn’t sleep or eat for a week. I also became irritable and easily annoyed … the experience was horrible.* (A1)

#### 4.2.4. Theme 4: relationships

##### 4.2.4.1. Desire for respect from faculty and clinical ward staff

The nursing students desired respect from their faculty members and ward staff but felt pressure to meet expectations and performance standards. They felt pressure to maintain a positive working relationship with these individuals.

*I felt like I had to work according to the ward staff’s preferences, as they seemed to like it when we could perform tasks quickly. Sometimes, I would have to skip some steps to please them, but I knew deep down that it wasn’t proper.* (D1)

*I would constantly think about the comments from the teachers and ward staff who said that I was too slow to learn. Their comments repeatedly emerged in my mind, and it made me unhappy.* (C2)

##### 4.2.4.2. Fear of disappointing parents

The nursing students expressed concern that they might let their family down if their academic performance was poor.

*I don’t know how to tell my parents if I failed my courses. They have high hopes for me … if they knew that I cannot not pass the exams and being expelled from the school, they [parents] would be very disappointed because of not only a waste of money for such a large amount of tuition fee but also their unfailing support and nurture to me …* (A1)

*I was afraid of telling my parents that I failed the course. They always thought that their daughter could someday become a nurse, and I was afraid of disappointing them.* (E1)

*I couldn’t allow myself to fail the program because it would cause a huge financial problem for my parents if I couldn’t get a job to pay back the student loan. I didn’t want to let them down.* (A2)

#### 4.2.5. Theme 5: pursuit of success and support

##### 4.2.5.1. Help seeking

Most nursing students used positive coping strategies to manage their stress. They sought help from friend and teachers to solve the academic and clinical learning problems.

*I sought advice from senior students and teachers for suggestions in course choices … [also] sought help from a teacher who taught me how to write care plan.* (E1)

*I will clarify the assessment requirements with the teacher if I have any queries.* (A2)

*I would ask the ward staff to observe my practice and give me advice during medication administration.* (A1)

##### 4.2.5.2. Self-reliance

The main strategy the nursing students used to handle stress and soothe their feelings was talking with friends. Engaging in artistic endeavors was another strategy.

*To calm myself down, I would listen to music, paint, or make dessert. I felt more comforted after doing these activities.* (D2)

*I would share my feelings with my friends and classmates because they understand my problems … I felt better after talking with them.* (C2)

*I would share my feelings with my friends and classmates because they understand my problems. I also practiced exercises and set some questions like the case scenarios provided by teachers in class. Then, I did the questions and asked the teachers for comments.* (C1)

*I vented my feelings to my friends and parents. I could talk about whatever I wanted, and I felt better after talking with them.* (B2)

## 5. Discussion

### 5.1. The sources of stress and change in stress levels

This study is the first longitudinal study to examine stress levels among nursing students in Hong Kong. The primary aim of this study was to explore the stressors of nursing students during their course of study. The findings of this study support previous research indicating that nursing students in Hong Kong experience stress during their course of study (Ching et al., 2020; Li and Hasson, 2020). The quantitative findings reveal that nursing students experienced higher levels of stress in their first and third years, where they perceived lower confidence and more personal problems, respectively. Nursing students who worked part-time for more than 10 h per week were found to have higher levels of psychological distress. The second year of study was associated with higher levels of psychological distress, which was linked to financial and time-related stress. In contrast, the fourth year of study was associated with academic and personal problems, which were perceived as more stressful. The qualitative data provided further insights into the stressors experienced by nursing students, which included academic performance issues, an unfavorable learning environment, coping challenges, and relationship issues. To manage their stress, nursing students utilized help-seeking and self-reliance strategies.

### 5.2. Stressful experience(s) of the nursing students

An examination of the study institution’s curriculum reveals that the high demand of the nursing courses offered in year 2 and the lengthy clinical practice (560 h) in year 4 likely contributed to these years being predictors of stress. Qualitative data showed that stress related to academic study arises not only from a heavy workload but also from students’ difficulty in understanding course information, assessment requirements, and a lack of resources to support learning. Furthermore, a tight study schedule hindered intellectual growth and curiosity, leading to unsatisfactory performance (Von Stumm et al., 2011; Cain, 2019; Chan et al., 2019). The fear of failure and lack of confidence that emerged were thus understandable. The lengthy clinical study period may have highlighted the overall lack of guidance and support provided by teachers. It is common for nursing students to experience high levels of pressure as they are constantly monitored and evaluated by nursing staff and teachers during their clinical learning (Bhurtun et al., 2019). Therefore, it is crucial to establish a supportive clinical environment that can help nursing students cope with the stressors they face.

The intense stress experienced by nursing students during their clinical studies was attributed not only to the busyness of the clinical environment but also to the frequent changes of clinical venues, which intensified adaptation demands. Stress also arose from the pressure to maintain positive relationships with tutors and clinical mentors. In contrast to Western culture, Confucianism, a prevalent cultural belief system in Hong Kong, emphasizes respect for authority, hierarchy, and collectivism (Tweed and Lehman, 2002). This cultural context may lead nursing students to be more deferential to their clinical instructors and teachers, which can impact their communication and assertiveness skills (Le, 2021).

Stressors related to personal issues and confidence were observed in years 1 and 3, respectively, which is consistent with previous studies conducted in different contexts (Sanad, 2019; Mazalová et al., 2022). In year 1, the transition from high school to college life can potentially amplify stress levels, and adjustment difficulties among freshmen entering university are often a contributing factor (Liu et al., 2019). They could be lack of confidence in looking after their patients due to lack of knowledge (Sanad, 2019). The qualitative data revealed that nursing students felt pressure to meet their parents’ expectations and feared disappointing them. The emphasis on filial piety, which highlights the importance of family relationships and obedience to parents and elders, is a core value in Chinese culture (Guo et al., 2021). This cultural context may help explain why, despite having an easier course load in year 3 compared to year 2, nursing students still experienced stress if they anticipated academic failure.

### 5.3. The relationships between stressors, coping strategies, stress and psychological distress

This study provides evidence of psychological distress experienced by nursing students throughout their studies. The predictors of psychological distress included financial and time constraints, as well as part-time job employment. Stressors related to finance and time were found to interact with year 2, while academic and personal problems were found to interact with year 4, contributing to psychological distress. Year 2 was characterized by packed courses, while year 4 was characterized by lengthy clinical study. The heavy workload and tight study schedule left little time for leisure activities and energy restoration, negatively impacting work-life balance and social life, which may have contributed to the personal problems expressed by nursing students. In addition, the nursing students reported physical and mental health issues such as sleep disturbance, loss of appetite, and depression. Coupled with these health problems, nursing students began to worry about their future in terms of job prospects and financial viability toward the end of their studies, further exacerbating their psychological distress.

Interestingly, stressors that predicted stress levels were not associated with psychological distress in the same year. This may be explained by nursing students’ adaptation to stress, which can decrease its destructive effects (Pavithra and Sivakumar, 2020). The coping behavior of nursing students is noteworthy; they rarely resorted to negative coping strategies but instead employed positive coping styles, such as seeking help and emotional support from friends, parents, and teachers. This is consistent with previous research, which has shown that nursing students tend to use problem-solving as their coping techniques (Bhurtun et al., 2019). Consistent with previous studies (Xu et al., 2020; Mazalová et al., 2022), nursing students reported a decrease in emotional problems toward the end of their studies, and psychological distress also declined over time. These decreases may be attributed to nursing students regaining confidence and adapting to academic study.

These findings highlight the need for nurse educators to thoughtfully design curricula that balance workload and assessments. Clear communication about learning objectives and assessment criteria can help avoid misunderstandings and improve engagement. To foster a positive learning environment, nurse educators should collaborate with clinical partners to create a culture of support and encouragement, which can include providing constructive and supportive feedback. As positive coping styles improve psychological functioning (Fornes-Vives et al., 2019), nursing programmes can provide workshops or classes on stress management and coping skills to help nursing students develop positive coping strategies. These sessions can inform students about the effects of stress on the body and mind, teach stress-reducing techniques like mindfulness and relaxation exercises, and promote healthy coping strategies.

To prevent or alleviate psychological distress, nursing schools could facilitate the formation of formal and informal peer support groups to foster a sense of community among students. In addition, nursing schools could provide students with resources for self-care, such as information on healthy eating, exercise, and sleep habits. By encouraging nursing students to prioritize their physical health, nursing schools can positively impact their mental health and ability to cope with stress.

### 5.4. Strengths and limitations of the study

The present study, which is the first longitudinal investigation into the experience of stress among nursing students in Hong Kong, offers valuable insights into the changes and trends in stress levels over time, and the long-term effects of stress on nursing students’ mental health and well-being. The findings of this study have important implications for nursing students in Hong Kong and other similar cultural contexts. By examining stress levels and stressors over time, this study provides a more comprehensive understanding of the impact of stressors at different stages of the academic journey. However, the study’s sample size was small, which may limit the generalization of the results. Nevertheless, the focus group interviews provided in-depth discussion of sensitive issues and causes of stress that may not have been detected by the closed questionnaires, and data saturation was achieved. Therefore, the findings may be useful in informing the development of interventions and support programs for nursing students in Hong Kong or in similar cultural contexts. Future studies could benefit from multisite research with larger sample sizes to increase the generalizability of the findings. Additionally, the study’s measurement of psychological distress only from the second year onward limits the interpretation of the results for the trends of psychological distress among all nursing students. Lastly, it is important to note that the data was collected prior to the COVID-19 pandemic, so the stressors expressed by nursing students may have been changed since then.

## 6. Conclusion

The results of this longitudinal study provide insights into the stressors faced by the nursing students and the ways they cope throughout their academic journey. The year of study is a predictor of stress levels among nursing students, with the first and senior years being the most stressful due to the heavy academic workload. Psychological distress was evident during their course of study, and nursing students who worked more part-time jobs tended to have higher levels of psychological distress. The junior year was associated with higher levels of psychological distress related to financial and time-related stress, while academic and personal problems were related to the senior year. The stressors that aggravated psychological distress varied across the course of study, highlighting the need for nurse educators to be sensitive to the needs of nursing students in different stages of their study when planning the curriculum. Addressing these challenges through clear communication, fostering a supportive learning environment, and providing resources and opportunities for students to develop positive coping strategies and maintain their physical health can help mitigate stress and psychological distress and enhance students’ overall well-being and success in their nursing education, preparing them for their roles as qualified nurses.

## Data availability statement

The raw data supporting the conclusions of this article will be made available by the authors, without undue reservation.

## Ethics statement

The studies involving humans were approved by Committee on the Use of Human and Animal Subjects in Teaching and Research (HASC) of Tung Wah College (HASC1415H02). The studies were conducted in accordance with the local legislation and institutional requirements. The participants provided their written informed consent to participate in this study.

## Author contributions

WC, PC, and MH: study conception, design, and data collection. WC, PC, FW, and MH: data analysis, interpretation, and critical revision of the article. WC: drafting of the article. All authors contributed to the article and approved the submitted version.

## Funding

This research study was funded by the College Research Grant (2016-00-51 CRG160402) of Tung Wah College. The publication fee of this study was funded by the Institutional Development Grant- Publication Caritas Institute of Higher Education (IDG-P220234).

## Conflict of interest

The authors declare that the research was conducted in the absence of any commercial or financial relationships that could be construed as a potential conflict of interest.

## Publisher’s note

All claims expressed in this article are solely those of the authors and do not necessarily represent those of their affiliated organizations, or those of the publisher, the editors and the reviewers. Any product that may be evaluated in this article, or claim that may be made by its manufacturer, is not guaranteed or endorsed by the publisher.
